# Impact of partial replacement of fish meal with polychaete meal (*Hediste diversicolor*) on growth, nutrient digestibility, fatty acid profiles, and fish-in: fish-out ratio in diets of European seabass, *Dicenctrarchus labrax*

**DOI:** 10.1007/s10695-026-01634-y

**Published:** 2026-01-28

**Authors:** Yasir Akbaş, Erkan Gümüş, Mehmet Ali Turan Koçer, Adem Kurtoğlu, İsa Aydın, Özgür Aktaş, Faruk Pak, Hüseyin Sevgili

**Affiliations:** 1 Department of Aquaculture, Faculty of Fisheries, Akdeniz University, 07058 Antalya, Türkiye; 2 Mediterranean Fisheries Research Production and Training Institute, Antalya, Türkiye; 3 Fisheries Application and Research Center & Department of Aquaculture, Eğirdir Fisheries Faculty, Isparta University of Applied Sciences, Eastern Campus, 32260 Isparta, Türkiye

**Keywords:** Fish meal, Fish oil, Polychaete meal, Fish in: fish out, European seabass

## Abstract

As polychaete meal (PM) from *Hediste diversicolor* has been reported to be a promising alternative to fish meal (FM) and fish oil (FO) in aquafeeds, we hypothesized that replacing FM with PM would support growth performance, feed utilization, and flesh fatty acid profile of European seabass (*Dicentrarchus labrax*). Therefore, this study was planned to investigate graded PM inclusion levels (0% [control], 5% [PM5], 10% [PM10], 15% [PM15] and 20% [PM20]), selected based on feasible incorporation limits for juvenile European seabass, as a partial substitute for FM (reduced from 29.45% in the control to 14.55 in PM20) in isonitrogenous (48% protein) and isolipidic (14% lipid) diets. A seven-week trial with a total of 150 fish (initial average weight of 14.56 ± 0.01 g) in triplicate tanks assessed growth performance, feed utilization, nutrient digestibility, body composition, fatty acid profiles, and fish-in-fish-out (FIFO) ratio. Growth performance, feed utilization, and organo-somatic indices of experimental fish remained unaffected by dietary PM levels (*P* > 0.05). A significant linear increase in apparent digestibility coefficients (ADCs) of protein, energy, and organic matter was observed with increasing PM inclusion (*P* < 0.05), while lipid ADCs remained unaffected *P* > 0.05). Whole-body protein and lipid content increased, with a corresponding decrease in moisture as dietary PM levels increased (*P* < 0.05). Fatty acid profiles and lipid quality indices of the liver and fillet were variably affected, liver PUFA levels declined with higher PM, whereas fillet long-chain PUFAs (Lc-PUFAs) such as arachidonic acid (ARA) and eicosapentaenoic acid (EPA) linearly increased. The FIFO ratio fell below 1 at a 20% PM inclusion. The findings suggest that PM can replace up to 50% of dietary FM and partly FO in seabass diets without compromising growth, nutrient utilization, or fillet quality while enhancing sustainability metrics.

## Introduction

Aquaculture production is increasing to meet rising demand for fish, elevating pressure on high-quality protein and lipid ingredients like fish meal (FM) and fish oil (FO) (Glencross et al. 2024a). These ingredients meet the amino acid and fatty acid requirements of cultured fish but face challenges of limited supply and high costs, necessitating the search for sustainable and cost-effective alternatives (Glencross et al. 2024a; Oliva-Teles et al. 2022; Pratiwy and Triyani 2022). Plant-based proteins, such as soybean meal, are widely available, but they contain anti-nutrients that can inhibit growth and nutrient utilization in fish (Gatlin et al. 2007; Muziri et al. 2022). Industrial rendering by-products and insect meals, such as *Hermetia illucens*, are being valorized as alternative protein sources, offering a sustainable solution to reduce dependency on FM (Siddiqua and Khan 2022; Thazeem et al. 2022). Animal-based proteins generally outperform plant-based proteins in terms of growth performance and feeding costs in fish (Hoerterer et al. 2022). Similarly, vegetable oils can partially replace FO without compromising growth or health in various fish species, but fillet fatty acid profiles can be distorted for human food (Seunghan et al. 2022; Turchini et al. 2009). Microalgae, fishery by-products, and krill can be refined to produce ingredients that allow the simultaneous replacement of dietary FM and FO (Idenyi et al. 2022; Oliva-Teles et al. 2022; Sevgi̇li̇ et al. 2012), suggesting that marine-based ingredients, particularly those produced by a circular method, have a high potential for use in aqua feeds (Eroldoğan et al. 2023).

The dietary use of polychaetes as a replacement for FM and FO in aquaculture diets has garnered little interest despite their high nutritional value and potential for sustainable aquaculture practices (Ballester‐Moltó et al. 2017; Leelatanawit et al. 2014; Mastoraki et al. 2023; Monteiro et al. 2024b; Rossi et al. 2025). The replacement of FM and FO with polychaetes in aquafeeds can mitigate the ecological and economic drawbacks associated with the over-reliance on wild-caught fish for these ingredients (Monteiro et al. 2024b; Pallab 2023). The inclusion of polychaete meal (PM) from *Alitta virens* in diets of European seabass up to 10% in place of FM yielded comparable growth and feed conversion efficiency (Monteiro et al. 2024b). The dietary PM was efficiently digested by meagre (*Argyrosomus regius*) unless it was added in excess of 10% (Mastoraki et al. 2023). The integration of polychaetes into aquaculture diets presents a viable strategy for reducing dependency on FM and FO, promoting sustainable and economically viable aquaculture practices (Shankar et al. 2023). Introducing polychaetes (*Nereis virens*) as an ingredient as a part of fresh materials, including mussels (*Mytilus platensis*) and 50% clams (*Chamelea galina* and *Paphia textile*) instead of commercial diets to gilthead seabream (*Sparus aurata*) growing from juvenile to commercial size yielded lower growth and nutrient utilization, but a supplementation of mineral premix to the fresh feed paid off significantly for the performance loss (Rossi et al. 2025). Dietary inclusion of PM from *A. virens* up to 10% to partially reduce FM in diets of juvenile European sea bass (*Dincentrarchus labrax*) resulted in comparable growth and feed efficiency with improved health, immune status, and antioxidant defenses (Monteiro et al. 2025). Still, efforts should be undertaken to gain further insight into the nutritional values of polychaetes in aquaculture diets.

Polychaete species such as *Hediste diversicolor* can upcycle nutrient-rich side streams from aquaculture and biogas production (e.g., fish sludge, uneaten feed, solid biogas digestate) into high-value biomass. When reared on these waste substrates, *H. diversicolor* typically attains 50–60% crude protein and 10–16% lipid (dry matter basis), with balanced essential amino acids and appreciable levels of long-chain n-3 PUFA, highlighting its suitability as a feed ingredient (Wang et al. 2019a, b; Malzahn et al. 2023; Anglade et al. 2023). Former studies focusing on integrating polychaete production on fish sludge and other side streams have demonstrated an efficient recovery of C, N, and P and reduced particulate waste discharge, thereby contributing to circular and more sustainable aquaculture (Nederlof et al., 2019; Wang et al. 2019a; Anglade et al. 2023; Santos et al. 2025). Consequently, PM derived from these waste-based cultures can offer a high-quality protein and oil source for carnivorous aquaculture fish.

Therefore, this study was planned to further explore the nutritional value of PM from *H. diversicolor* for European seabass based on selected variables, including growth, nutrient utilization, apparent nutrient and fatty acid digestibility coefficients, fatty acid compositions, lipid qualities, and estimated elongase and desaturase activities in liver and fillet, as well as fish in: fish out ratio (FIFO).

## Materials and methods

The experiment was carried out in the experimental unit of the Demre Campus of the Mediterranean Fisheries Research, Production and Training Institute Directorate (MEDFRI), Antalya, Türkiye.

### Experimental diets

Polychaete (*Hediste diversicolor*) was collected from the Aegean Sea and delivered live on ulva leaves or frozen in styrofoam boxes to the MEDFRI fish nutrition laboratory. Upon arrival, the live polychaetes were cleaned, thoroughly cleaned with fresh water and stored at −20°C until use. The live-delivered polychaete was freeze-dried (Crydos-50, Telstar Industrial, Terrassa, Spain), whereas the frozen-delivered polychaete was dried at 65 °C overnight. Both materials were finely ground using a high-speed grinder (Henan Shuoman Machinery Equipment Co., Ltd., Henan, China) and then mixed equally. The comparative nutritional composition of PM and a mixture of FM is given in Table 1. The PM was incorporated into isoproteic (48%) and isolipidic (14%) diets at 0%, 5%, 10%, 15% and 20% at the expense of FM and FO (Table 2). Chromic oxide was also included in the diets as an indigestible marker for the estimation of apparent nutrient digestibility coefficients. The ingredients were thoroughly mixed, and then deionized water was added until a dough-like consistency was achieved. The mixtures were extruded using a meat chopper with a die of 2 mm diameter. The resulting pellets were broken into small pieces, dried at 65 °C, and stored in airtight bags until used.

**Table 1:** Nutrient compositions of fish meal mixture and polychaete meal used in the experiment (% dry matter basis)

Nutrients	FM	PM	Requirement
Dry matter	92.9	90.2	
Protein	76.8	59.4	
Crude lipid	7.1	5.9	
Crude ash	14.0	14.6	
(MJ/kg)	21.3	19.8	
Fatty acids (% of total fatty acids)			
14:0	6.30	1.25	
15:0	0.58	0.72	
16:0	29.4	17.0	
17:0	0.58	0.84	
18:0	10.9	13.5	
20:0	0.44	0.27	
21:0	0.05	ND	
22:0	0.18	ND	
24:0	0.89	0.38	
∑SFA	49.2	34.2	
16:1	7.23	3.78	
17:1	0.15	0.52	
18:1n-9	11.7	7.34	
18:1n-7	3.69	3.12	
20:1	0.73	0.78	
22:1n-9	0.57	0.67	
24:1	0.08	1.63	
∑MUFA	23.9	17.8	
18:2n-6	1.83	0.93	
C8:3n-3	1.32	5.04	
18:4n-3	0.94	0.91	
20:2n-6	0.19	1.30	
20:3n-6	0.94	0.30	
20:4n-6 (ARA)	0.19	0.93	
22:2n-6	0.50	3.66	
20:5n-3 (EPA)	6.42	16.60	
22:4n-6	0.20	0.28	
22:5n-3	0.61	1.23	
22:6n-3 (DHA)	7.38	7.33	
∑PUFA	20.1	38.5	
PUFA/SFA	0.49	1.13	
∑n6	3.38	7.38	
∑n3	16.7	31.1	
Lc-n-3 PUFA	14.4	25.2	
Lc-n-6 PUFA	2.02	5.16	
∑n6/∑n3	0.24	0.24	
DHA/EPA	1.53	0.44	
Amino acids^1^			Requirement^2^
Essential			
Arginine	4.05	3.59	2.79
Histidine	1.76	1.55	0.97
Isoleucine	2.70	2.75	1.58
Leucine	5.06	4.37	2.61
Lysine	5.43	4.90	2.91
Methionine + cysteine	2.17	2.45	1.39
Phenylalanine + tyrosine	5.12	4.07	1.58
Threonine	2.92	1.85	1.64
Valine	3.16	2.39	1.76
Tryptophan	0.64	0.24	0.36
Non-essential			
Alanine	4.35	5.98	
Aspartic acid	5.25	4.72	
Glutamic acid	8.49	7.48	
Glycine	4.02	3.47	
Serine	2.68	3.59	
Taurine	0.41	0.60	0.2^3^

^1^Fish meal amino acid contents are average for fish meals of Danish, Domestic, and Peruvian (Sevgili et al. 2015). PM amino acid contents were taken from wild polychaete (Seekamp 2017)

^2^Calculated from the amino acid requirement from Kaushik (1998)

^3^NRC (1993)

∑SFA: total saturated fatty acids, ∑MUFA: total monounsaturated fatty acids, ∑PUFA: total polyunsaturated fatty acids, ∑n-6: total n-6 polyunsaturated fatty acids, ∑n-3: total n-3 polyunsaturated fatty acids, Lc PUFA: long-chain polyunsaturated fatty acids, (ARA): arachidonic acid, EPA: eicosapentaenoic acid, DHA: docosahexaenoic acid

**Table 2:** Ingredient and nutrient compositions of experimental diets of European sea bass

Ingredients	Control	PM5	PM10	PM15	PM20
FM	29.45	25.72	22.00	18.27	14.55
PM	0.00	5.00	10.00	15.00	20.00
Soybean meal^1^	10.50	10.50	10.50	10.50	10.50
Corn gluten meal^1^	7.50	7.50	7.50	7.50	7.50
Wheat gluten^1^	4.00	4.00	4.00	4.00	4.00
Poultry by-product meal^1^	9.67	9.67	9.67	9.67	9.67
Soybean protein concentrate^1^	2.50	2.50	2.50	2.50	2.50
FO^1^	10.05	9.92	9.78	9.65	9.52
Wheat flour^1^	24.28	22.84	21.49	20.14	18.80
Mono hydrogen phosphate	0.25	0.56	0.76	0.96	1.17
Vitamin premix^2^	0.50	0.50	0.50	0.50	0.50
Mineral premix^3^	0.10	0.10	0.10	0.10	0.10
Choline chloride	0.20	0.20	0.20	0.20	0.20
Carboxymethyl cellulose	0.50	0.50	0.50	0.50	0.50
Chromic oxide	0.50	0.50	0.50	0.50	0.50
Nutrient concentrations (dry matter basis %)					
Dry matter	95.8	95.7	95.5	95.6	95.5
Protein	50.1	50.0	49.6	49.1	48.9
Lipid	14.9	14.3	14.5	14.2	14.4
Ash	8.7	9.2	9.6	9.9	9.9
Gross energy (MJ/kg)	22.2	22.0	22.0	21.8	21.8
Digestible energy (MJ/kg)^4^	16.9	16.4	16.8	17.0	17.4

^1^Provided from Akvatek Aquaculture Inc., İzmir, Türkiye

^2^Vitamin mixture contains in each kg of diet, 4.000 IU vitamin A, 400 IU vitamin D3, 40 mg vitamin E, 3 mg vitamin K3, 4 mg vitamin B1, 6 mg vitamin B2, 40 mg niacin, 10 mg Cal D-Pantothenate, 4 mg vitamin B6, 10 mg vitamin B12, 100 mg D-Biotin, 1.2 mg folic acid, 40 mg vitamin C, 60 mg inositol

^3^Mineral mixture contains in each kg of diet, 50 mg manganese, 50 mg zinc, 10 mg copper, 150 mg cobalt, 800 mg iodine, 150 mg selenium, and 50 mg iron

^4^Calculated based on the apparent digestibility coefficients of energy (see Sections "Calculations" and "Chemical analysis")

### Experimental conditions

Juvenile European sea bass (D. *labrax*) procured from Kılıç Holding, Muğla, Türkiye, were transferred using a fish transportation truck to the experimental unit of Demre Campus of the MEDFRI. They were acclimated to experimental conditions for 4 weeks in 400 L tanks. Fish with an initial average weight of 14.56 ± 0.01 g were randomly allocated to triplicate tanks (10 fish/tank) per diet. Fish were fed to apparent satiation twice a day at 09:00 and 15:00 for seven weeks under a 12L:12D photoperiod. Uneaten pellets were siphoned 30 min after each meal and pellet counts were recorded to determine net feed consumptions. The experimental tanks were connected to a flow-through system using full-strength seawater. Water flow to each experimental tank was about 5 L per minute. Water quality parameters, including temperature, dissolved oxygen, pH, and salinity, were recorded daily using a handheld device. The average water quality variables, including water temperature, dissolved oxygen, pH and salinity over the experiment, were 21.07 ± 0.17°C, 8.08 ± 0.05 mg/L, 8.00 ± 0.03, and 38.63 ± 0.05 mg/L, respectively. No fish died during the experiment.

### Samplings

Fecal samples were collected starting from the third week onwards to determine apparent nutrient digestibility coefficients (ADC) of nutrients. The samples were taken three days a week by siphoning the intact fish feces in the mornings before the first meal. The collected samples from each tank were washed with deionized water, stored at −20°C and pooled by tank before analysis. Initial whole-body composition was determined from 20 fish euthanized with an overdose of anesthesia (1.2 mL/L phenoxyethanol), and frozen at −20°C until analysis. At the end of the experiment, four fish from each tank (*n* = 12 per treatment) were sampled to determine body indices (condition factor, CF; viscero-somatic index, VSI; and hepato-somatic index, HSI) after the overdose of anesthesia. The liver and muscles of the same fish were separated and kept at −20°C until fatty acid analysis. Moreover, four fish per tank were also sampled and stored whole at −20°C for the determination of final whole body compositions.

### Calculations

#### Growth and nutrient utilization variables

Growth and feed utilization parameters were calculated according to formulas given below: Weight gain (WG g/fish) = W_t_–W_0_,

Specific growth rate (SGR %/day) = 100(ln W_t_ln W_0_)/t,

Feed conversion ratio (FCR) = dry feed intake (g)/wet weight gain (g),

Daily feed intake (DFI g/kg MBW/day) = (dry matter intake/MBW^0.8^)/day.

Metabolic body weight (MBW) = (Geometric mean of initial weight (IW) and final weight (FW))^0.8^

Protein efficiency rate (PER) = weight gain (g) /protein fed (g),

Condition factor (CF) = 100 (Wt (g) /L^3^(cm)),

Viscero-somatic index (VSI %) = 100 (visceral weight (g)/body weight (g)),

Hepato-somatic index (HSI %) = 100 (liver weight (g)/body weight (g)),

Nutrient (N, lipid, energy) intake (g or kJ/kg ABW/day) = (nutrient intake (g or kJ/kg ABW))/days,

Nutrient (N, lipid, energy) gain (g or kJ/kg ABW/day) = ((final body nutrient content (g or kJ)–initial body nutrient content (g or kJ))/kg ABW)/days,

Nutrient (N, lipid, energy) retention = 100 (nutrient gain (g or kJ)/nutrient intake (g or kJ),

W_t_ (g) is the fish body weight at day t and W0 at day 0, t (days), is the duration of the experiment, ABW is the average body weight of fish at W_t_ and W_0_, L is the total length (cm).

Solid nitrogen (Solid N) (g/kg fish) = ((N in ingested feed (DM)) × ((1 – ADC of N) × (N content of feed))/WG (kg).

Dissolved nitrogen (Dissolved N) (g/kg fish) = ((N in feed × ADC of N) − (N retention))/WG (kg) Total nitrogen (Total N) (g/kg fish) = ((Solid N g/kg fish) + (Dissolved N g/kg fish))/WG(kg).

Total solid (g/kg fish) = ((ingested feed (DM)) × (1 − (ADC DM of feed))/WG(kg).

Fish in/fish out ratio (FIFO) (kg feed fish used/kg European seabass produced) = FM used in diet (kg) FCR × 100/22.5 (Tacon and Metian 2008).

#### Apparent nutrient digestibility

Nutrient ADCs of test diets for crude nutrients and fatty acids (based on the absolute quantities) were calculated according to the formula suggested by NRC (2011).

$${\text{Dry matter ADC}}_{test \;diets}=100 \left[1-\left({{Cr}_{2}{O}_{3}}_{feed}/{{Cr}_{2}{O}_{3}}_{feces}\right)\right]$$

$${\text{Nutrient ADC}}_{test \;diets}=100 \left[1-\left(\frac{{{Cr}_{2}{O}_{3}}_{feed}}{{{Cr}_{2}{O}_{3}}_{feces}}\times \frac{{Nutrient}_{feces}}{{Nutrient}_{feed}}\right)\right]$$

#### Lipid quality indices of liver and fillets

The indices of lipid quality of liver and fillet of fish were calculated using the formula below:

-  *Atherogenic index (AI)=(C*12:0 + (4 × *C*14:0) + (*C*16:0))/(Σ*MUFA *+ Σ*n – 6PUFA *+ Σn – 3*PUFA*) (Ulbricht and Southgate 1991).
- *Thrombogenic index (TI)* = ((*C*14:0 + *C*16:0 + *C*18:0)/((0,5 × Σ*MUFA*+ 0,5 × Σ*n* – 6*PUFA *+ 3 × Σ*n* – 3*PUFA*)+((Σ*n* – 3*PUFA*)/(Σ*n* – 6*PUFA*))) (Ulbricht and Southgate 1991)
- Hypocholesterolemic/hypercholesterolemic ratio (h/H) = ((C18:1n9 + C18:2n6 + C20:4n6 + C18:3n3 + C20:5n3 + C22:5n3 + C22:6n3)/(C14:0 + C16:0))(Santos-Silva et al. 2002).
- *Peroxidation index (PI)* = 0.025 × (%Σ *monoenoic*) + 1 × (%Σ *dienoic*) + 2 × (%Σ *trienoic*) + 4 × (%Σ *tetraenoic*) + 6 × (%Σ *pentaenoic*) + 8 × (%Σ *hexaenoic*) (Witting and Horwitt 1964).

#### Elongase and desaturase activity in liver and fillet

Elongase and desaturase activities were not measured directly, but were calculated indirectly from product/precursor ratios of fatty acids in liver and fillet, as followed by Mattioli et al. (2018).

1. Thioesterase = C16:0/C14:0;
2. Elongase = C18:0/C16:0;
3. Δ9 desaturase (C16) = [(C16:1)/(C16:1 + C16:0)] × 100;
4. Δ9 desaturase (C18) = [(C18:1)/(C18:1 + C18:0)] × 100;
5. Δ9 desaturase (C16 + 18) = [(C16:1 + C18:1)/(C16:1 + C16:0 + C18:1 + C18:0)] × 100;
6. Δ5 + Δ6 desaturase (n6) = [(C20:2n-6 + C20:4n-6)/(C18:2n-6 + C20:2n-6 + C20:4n-6)] × 100;
7. Δ5 + Δ6 desaturase (n3) = [(C20:5n-3 + C22:5n-3 + C22:6n-3)/(C18:3n-3 + C20:5n-3 + C22:5n-3 + C22:6n-3)] × 100.

### Chemical analysis

The fecal samples were dried at 65 °C, ground and screened through 500 µm to separate scales before the nutrient analysis. Proximate analysis, except crude lipid, of experimental diets, ingredients, fish, and feces were performed according to the methods of (AOAC 1990): dry matter at 104 °C till constant weight, ash content by incineration in a muffle furnace at 600 °C for 2 h; crude protein (N × 6.25) by the Dumas method using a Dumas Nitrogen Analyzer (Velp NDA 701-Monza, Brianza-Italy). Crude lipid analysis was performed using a fast lipid extraction system (ANKOMXT15 Extractor, ANKOM Technology, Macedon, USA). Gross energy values of ingredients, diets, and feces were calculated using conversion factors of 39.5, 23.6, and 17.2 MJ/kg for fat, protein, and carbohydrates (NRC 2011). Chromic oxide analysis was carried out according to the method described by Furukawa and Tsukahara (1966). Total lipids from liver and muscle samples were extracted according to Bligh and Dyer (1959), and fatty acid methyl esters (FAME) were prepared as described by Ichihara et al. (1996). FAME profiles were determined using a gas chromatograph (Focus GC, Thermo Electron, Waltham, MA) equipped with an auto sampler, FID detector, and fused-silica capillary column (30 m × 0.32 mm ID × 0.25 μm film). The oven temperature was programmed from 140 °C (5 min) to 200 °C at 4 °C min⁻^1^, and then to 220 °C at 1 °C min⁻^1^; injector and detector temperatures were 220 °C and 280 °C, respectively. Peaks were identified by comparison with a SUPELCO FAME standard (Sigma-Aldrich).

### Statistical analysis

The response variables obtained from the study were checked for normality and homogeneity of variances using the Shapiro–Wilk and Bartlett tests, respectively, before analysis of variance and polynomial contrasts. The Tukey–Kramer HSD test was used to discriminate between the treatments that were significantly different. Linear and quadratic polynomial contrasts were used to detect the effects of various dietary PM on the variables using the GLM procedure in JMP software (version 8, SAS Institute Inc., Cary, NC). A significance level of *P* < 0.05 was considered in statistical analyses, with a level of *P* ≤ 0.1 indicating a significant tendency.

## Results

### Nutrient composition of PM

PM had lower protein, lipid, ∑SFA, ∑MUFA, DHA/EPA and several amino acids (arginine, histidine, leucine, lysine, phenylalanine + tyrosine, threonine, valine and tryptophan) than FM, but comparable or higher ash, 20:4n-6 (ARA), EPA, DHA, ∑PUFA, ∑n3, ∑n6, PUFA/SFA, ∑n6/∑n3, Lc-n-3 PUFA, Lc-n-6 PUFA and certain amino acids including isoleucine, methionine + cysteine, and taurine (Table 1 and Table 3).

**Table 3:** Fatty acid compositions of experimental diets

Fatty acids	Control	PM5	PM10	PM15	PM20
C14:0	2.29	2.23	2.06	1.95	1.88
C15:0	0.18	0.18	0.18	0.18	0.18
C16:0	14.5	14.3	14.1	14.0	14.4
C17:0	0.22	0.20	0.20	0.21	0.21
C18:0	5.41	5.36	5.66	5.58	6.02
C20:0	0.10	0.11	0.11	0.11	0.09
C21:0	0.17	0.17	0.16	0.16	0.15
C22:0	0.17	0.18	0.17	0.17	0.17
C24:0	0.33	0.32	0.30	0.28	0.25
∑SFA	23.4	23.0	22.0	22.6	23.4
C16:1	3.02	2.97	2.92	2.90	2.90
C17:1	0.10	0.14	0.10	0.10	0.05
C18:1n-9	32.7	33.0	32.5	32.1	31.8
C:181n-7	3.30	3.22	3.31	3.38	3.46
C20:1	4.10	4.20	4.23	4.25	4.29
C22:1n-9	0.39	0.34	0.38	0.36	0.32
C24:1	0.13	0.14	0.11	0.15	0.17
∑MUFA	43.8	44.0	43.5	43.2	43.0
C18:2n-6	17.1	17.1	16.9	17.3	17.5
C18:3n-3	2.59	2.54	2.60	2.60	2.62
C18:4n-3	0.59	0.61	0.62	0.63	0.62
C20:2n-6	0.71	0.72	0.73	0.73	0.74
C20:3n-6	1.38	1.34	1.35	1.32	1.26
C20:4n-6 (ARA)	0.31	0.18	0.33	0.35	0.23
C22:2n-6	0.46	0.45	0.47	0.45	0.47
C20:5n-3 (EPA)	2.47	2.57	2.72	2.91	2.98
C22:4n-6	0.11	0.12	0.13	0.13	0.12
C22:5n-3	0.83	0.85	0.96	0.92	0.95
C22:6n-3 (DHA)	2.95	2.97	3.07	3.15	3.05
∑PUFA	29.5	29.4	29.9	30.5	30.6
PUFA/SFA	1.26	1.28	1.30	1.34	1.31
∑n6	20.0	19.9	19.99	20.2	20.3
∑n3	9.43	9.54	9.97	10.2	10.2
n6/n3	2.13	2.08	2.00	1.98	1.99
DHA/EPA	1.19	1.16	1.13	1.08	1.02

*∑SFA*: total saturated fatty acids, *∑MUFA*: total monounsaturated fatty acids, *∑PUFA*: total polyunsaturated fatty acids, *∑n-6*: total n-6 polyunsaturated fatty acids, *∑n-3*: total n-3 polyunsaturated fatty acids, *Lc PUFA*: long-chain polyunsaturated fatty acids, *(ARA)*: arachidonic acid, *EPA*: eicosapentaenoic acid, *DHA*: docosahexaenoic acid

### Growth performance and feed utilization

Juvenile European seabass fed diets with increasing levels of PM showed comparable growth and nutrient utilization performance among the different diets (*P* > 0.05) (Table 4). Fish tended to consume more feed as dietary PM levels increased (P_*LINEAR*_ = 0.068). Organo-somatic indices and CF values of fish were comparable among the treatments (*P* > 0.05) (Table 4).

**Table 4:** Growth, nutrient utilization and organ indices of European seabass fed increasing PM levels for 49 days*

	Control	PM5	PM10	PM15	PM20	Pooled SEM	*P* value		
							ANOVA	Linear	Quadratic
IW (g/fish)	14.6	14.6	14.6	14.6	14.5	0.03	-	-	-
FW (g/fish)	34.7	32.5	31.4	36.2	33.3	2.50	0.700	0.900	0.604
SGR (%/day)	2.06	1.99	1.89	2.21	1.94	0.25	0.900	0.970	0.970
FCR	1.27	1.41	1.46	1.35	1.42	0.11	0.767	0.421	0.384
DFI (g/kg MBW/day)	10.7	10.8	10.8	11.9	11.3	0.38	0.217	*0.068*	0.801
PER	1.58	1.45	1.39	1.54	1.45	0.11	0.756	0.591	0.390
CF (%)	1.13	1.12	1.13	1.09	1.11	0.04	0.902	0.359	0.896
VSI (%)	10.1	10.2	10.6	10.0	9.86	0.37	0.739	0.481	0.249
HSI (%)	2.21	2.22	2.19	2.16	2.13	0.09	0.955	0.364	0.815

Control (0% PM), PM5 (5% PM), PM10 (10% PM), PM15 (15% PM), and PM20 (20% PM), where PM denotes polychaete meal from *H. diversicolor*

^*^Significant P values less than 0.05 are shown bold, whereas those significant trends between 0.05 and 0.10 are shown in Italic and underlined

*IW*; initial weight, *FW*; final weight, *SGR*; specific growth rate, *FCR*; feed conversion ratio, *DFI*; daily feed intake, *MBW*; metabolic body weight, *PER*; protein efficiency rate, *CF*; condition factor, *VSI*; viscero-somatic index, *HSI*; hepato-somatic index

The effects of dietary incorporation of PM on nutrient utilization data are displayed in Table 5. Dietary intakes of N, lipid, and energy were not influenced by PM levels (*P* > 0.05). On the other hand, the lipid gain of fish linearly increased in parallel with dietary levels of PM (P_*LINEAR*_ = 0.018). A similar trend, but with a significant tendency, was observed in the energy gain in fish (P_*LINEAR*_ = 0.075). Dietary inclusion of PM did not significantly change N and energy retentions, total solid wastes, and solid and dissolved N losses (*P* > 0.05) (Table 5).

**Table 5:** Nutrient utilization of European sea bass fed increasing levels of dietary PM*

Nutrients	Control	PM5	PM10	PM15	PM20	Pooled SEM	P value		
							ANOVA	Linear	Quadratic
***Intake***									
N (g/kg MBW/day)	0.86	0.87	0.85	0.93	0.88	0.03	0.365	0.192	0.767
Lipid (g/kg MBW/day)	1.59	1.55	1.56	1.69	1.62	0.06	0.451	0.248	0.792
Energy (kJ/kg MBW/day)	238	238	237	260	246	8.35	0.326	0.144	0.901
***Gain***									
N (g/kg MBW/day)	0.23	0.21	0.21	0.25	0.24	0.03	0.755	0.378	0.487
Lipid (g/kg MBW/day)	1.27	1.25	1.42	1.50	1.52	0.11	0.311	**0.018**	0.989
Energy (kJ/kg MBW/day)	83.5	79.6	86.2	95.9	95.1	7.8	0.530	*0.075*	0.707
***Retention***									
N (%)	26.2	23.9	24.0	26.5	26.8	2.7	0.882	0.580	0.367
Lipid (%)	79.9	80.8	90.8	89.0	93.6	6.2	0.459	**0.043**	0.826
Energy (%)	35.0	33.6	36.5	37.0	38.5	2.7	0.753	0.163	0.673
***Nutrient wastes***									
Solid N (g/kg WG)	12.6	14.0	13.7	11.7	11.3	1.4	0.600	0.201	0.232
Dissolved N (g/kg WG)	62.9	72.4	74.8	67.4	70.6	8.1	0.856	0.630	0.386
Total N (g/kg WG)	75.5	86.4	88.4	78.9	81.6	9.45	0.862	0.848	0.356
Total solids (g/kg WG)	429	500	478	415	401	39.98	0.399	0.211	0.119

Control (0% PM), PM5 (5% PM), PM10 (10% PM), PM15 (15% PM), and PM20 (20% PM), where PM denotes polychaete meal from *H. diversicolor*

^*^Significant *P* values less than 0.05 are shown bold, whereas those significant trends are shown in Italic and underlined

*MBW*; metabolic body weight, *WG*; weight gain

### Nutrient and fatty acid digestibility

The inclusion of PM in diets in place of FM resulted in a significant linear increase in the ADC of dry matter (*P* < 0.05) (Table 6). A similar significant increase was also detected in ADCs for protein, energy, and organic matter as dietary PM increased (P_*LINEAR*_ = 0.001). The ADCs of lipid were comparable regardless of dietary PM levels (*P* > 0.05).

**Table 6:** Apparent nutrient digestibility coefficients of experimental diets fed to European sea bass*

Nutrients	Control	PM5	PM10	PM15	PM20	Pooled SEM	P value		
							ANOVA	Linear	Quadratic
Dry matter (%)	66.2^bc^	64.6^c^	67.3^bc^	69.5^ab^	71.6^a^	0.91	**0.002**	**0.001**	**0.037**
Lipid (%)	94.0	93.9	93.9	93.4	95.2	0.47	0.154	0.155	*0.052*
Protein (%)	87.7^b^	87.7^b^	88.3^ab^	89.3^ab^	90.1^a^	0.50	**0.022**	**0.001**	0.234
Energy (%)	75.9^bc^	74.4^c^	76.6^abc^	77.9^ab^	79.7^a^	0.69	**0.003**	**0.001**	0.269
Organic matter (%)	69.7^bc^	67.7^c^	70.6^abc^	72.4^ab^	74.4^a^	0.85	**0.003**	**0.001**	**0.036**

^*^Values with different superscripts at the same row are significantly different from each other (*P* < 0.05). Significant P values less than 0.05 are shown bold, whereas those significant trends between 0.05 and 0.10 are shown in Italic and underlined

Control (0% PM), PM5 (5% PM), PM10 (10% PM), PM15 (15% PM), and PM20 (20% PM), where PM denotes polychaete meal from *H. diversicolor*

The apparent fatty acid digestibility values of the experimental diets are shown in Table 7. Total SFA digestibility quadratically increased with increasing dietary PM levels (P_*LINEAR*_ and P_*QUADRATIC*_ < 0.05). Although some individual quadratic effects of dietary PM levels on MUFAs (C:18:1n-7 and C22:1n-9) were observed, no significant change was observed in ADC of ∑MUFA among the diets (*P* > 0.05). Individual n-6 and n-3 PUFAs were similarly digested by fish fed with increasing dietary PM levels (*P* > 0.05). However, the ADCs of Lc-PUFA significantly increased as dietary PM levels increased (P_*LINEAR*_ = 0.022). The ADCs of total FAs were quadratically affected by the increasing dietary PM levels (P_*QUADRATIC*_ = 0.030).

**Table 7:** Apparent digestibility coefficients of fatty acids of experimental diets for European seabass (%)^1^

Fatty acids	Control	PM5	PM10	PM15	PM20	Pooled SEM	P values		
							ANOVA	Linear	Quadratic
14:0	94.3	94.4	93.8	94.0	94.2	0.69	0.972	0.756	0.652
15:0	93.8	94.3	94.6	95.9	95.0	1.29	0.835	0.256	0.645
16:0	87.2	86.2	86.3	85.6	88.9	0.80	0.102	0.224	**0.010**
17:0	90.4	90.3	90.9	90.4	94.3	1.54	0.358	0.*071*	0.176
18:0	64.4^b^	60.2^b^	65.5^b^	63.3^b^	74.9^a^	2.02	**0.005**	**0.002**	**0.006**
20:0	61.2	66.2	62.8	65.3	74.5	3.45	0.137	**0.018**	0.229
21:0	100	100	100	100	100	-	-		
22:0	81.3	83.0	78.8	78.4	86.9	2.45	0.176	0.373	**0.063**
24:0	94.0	94.3	92.8	92.9	96.2	0.89	0.116	0.264	**0.024**
∑SFA	82.7^ab^	81.5^ab^	82.0^ab^	81.0^b^	86.0^a^	1.03	**0.042**	**0.064**	**0.008**
16:1	96.3	96.4	95.6	96.1	96.7	0.55	0.681	0.674	0.190
17:1^2^	−269	−245	−120	−324	−242	42.5	0.059	0.869	0.252
18:1n-9	98.3	98.4	98.1	98.2	98.6	0.23	0.622	0.448	0.184
181n-7	96.1	96.3	95.8	96.2	97.1	0.57	0.585	0.218	**0.190**
20:1	100	100	100	100	100	-	-	-	-
22:1n-9	96.6	95.6	95.0	96.0	96.8	0.46	*0.093*	0.525	**0.002**
24:1	89.5	91.2	86.6	89.4	91.8	2.00	0.439	0.639	0.208
∑MUFA	97.6	97.4	97.2	97.0	97.6	0.31	0.540	0.604	0.109
18:2n-6	97.5	97.5	97.3	97.4	98.0	0.34	0.600	0.314	0.159
C8:3n-3	97.8	97.6	97.8	97.3	98.4	0.31	0.173	0.311	*0.068*
18:4n-3	92.1	91.7	91.1	90.5	94.0	1.24	0.393	0.447	*0.069*
20:2n-6	98.6	98.6	98.7	98.6	99.0	0.18	0.421	*0.064*	0.308
20:3n-6	100	100	100	100	100	-	-	-	-
20:4n-6 (ARA)	100	100	100	100	100	-	-	-	-
22:2n-6	100	100	100	100	100	-	-	-	-
20:5n-3 (EPA)	98.3	97.9	98.3	97.8	99.0	0.27	*0.086*	0.144	*0.058*
22:4n-6	82.0	87.5	94.5	88.2	95.1	4.57	0.304	*0.051*	0.496
22:5n-3	99.4	99.2	100	100	100	0.47	0.574	0.115	0.791
22:6n-3 (DHA)	97.7	98.0	97.9	98.1	98.4	0.37	0.744	0.130	0.823
∑PUFA	97.7	97.7	97.7	97.6	98.3	0.30	0.445	0.162	0.158
TOTAL	94.2	94.0	94.0	93.6	95.3	0.43	0.129	0.166	**0.030**
∑n-6	97.7	97.8	97.7	97.7	98.3	0.30	0.550	0.223	0.189
∑n-3	97.7	97.6	97.8	97.5	98.5	0.32	0.301	0.107	0.139
LC PUFA	98.4	98.4	98.7	98.5	99.1	0.20	0.185	**0.022**	0.427

^1^Values with different superscripts in the same row are significantly different from each other (*P* < 0.05). Significant P values less than 0.05 are shown bold, whereas those significant trends between 0.05 and 0.10 are shown in Italic and underlined

Control (0% PM), PM5 (5% PM), PM10 (10% PM), PM15 (15% PM), and PM20 (20% PM), where PM denotes polychaete meal from *H. diversicolor*

*∑SFA*: total saturated fatty acids, *∑MUFA*: total monounsaturated fatty acids, *∑PUFA*: total polyunsaturated fatty acids, *∑n-6*: total n-6 polyunsaturated fatty acids, *∑n-3*: total n-3 polyunsaturated fatty acids, *Lc-PUFA*: long-chain polyunsaturated fatty acids, *(ARA)*: arachidonic acid, *EPA*: eicosapentaenoic acid, *DHA*: docosahexaenoic acid

^2^Negative ADC values occasionally occur for trace fatty acids present at very low dietary levels (≤ 0.14% of total fatty acids for 17:1 in this study). Small analytical differences between diet and feces at such low concentrations can mathematically produce negative digestibility outcomes, which should therefore be interpreted as analytical noise rather than true biological effects

### Whole body composition

There was a linear decrease in the whole-body moisture with increasing dietary PM concentrations, whereas a significant linear increase was observed for whole-body protein and lipid (P_*LINEAR*_ < 0.05) (Table 8).

**Table 8:** The whole-body proximate compositions of sea bass fed varying levels of PM*

Nutrients	Initial	Control	PM5	PM10	PM15	PM20	Pooled SEM	P value		
								ANOVA	Linear	Quadratic
Moisture (%)	69.2	66.6	66.3	64.6	65.2	64.3	*0.57*	*0.055*	**0.003**	0.538
Protein (%)	15.5	16.1	16.0	16.5	16.5	17.1	0.44	0.443	**0.039**	0.464
Lipid (%)	8.85	12.4	12.8	14.4	13.5	14.4	0.58	0.108	**0.010**	0.487
Ash (%)	4.00	3.95	3.91	4.00	3.89	3.97	0.11	0.949	0.914	0.889

Control (0% PM), PM5 (5% PM), PM10 (10% PM), PM15 (15% PM), and PM20 (20% PM), where PM denotes polychaete meal from *H. diversicolor*

^*^Significant P values less than 0.05 are shown bold, whereas those significant trends between 0.05 and 0.10 are shown in Italic and underlined

### Fatty acid profiles and lipid quality indices of liver and fillet

#### The liver

The 16:0, 18:0, and ∑SFA levels of the liver linearly increased with dietary PM concentrations (P_*LINEAR*_ < 0.05) (Table 9). Despite significant linear reductions in 17:1, 20:1, and 22:1n-9 with PM levels (P_*LINEAR*_ < 0.05), the sum of MUFAs remained unaffected. Liver PUFAs, including 18:2n-6, 18:4n-3, 20:3n-6, 20:4n-622:2n-6, 20:5n-3, and 22:6n-3, reduced linearly with increasing dietary PM inclusion levels (P_*LINEAR*_ < 0.05). Lipid quality indices and fatty acid-related enzyme activities in the liver of fish fed experimental diets are shown in Table 10. The PI of experimental treatments improved linearly with dietary PM levels (P_*LINEAR*_ = 0.006). Thioesterase and ∆5 + ∆6 desaturase (n-6) activities of the liver of fish fed increasing PM levels linearly increased, whereas ∆9 desaturase (C16), ∆9 desaturase (C18), ∆9 desaturase (C16 + C18), and ∆5 + ∆6 desaturase (n-3) significantly decreased (P_*LINEAR*_ < 0.05).

**Table 9:** Fatty acid profiles of liver of European sea bass fed experimental diets for 49 days*

Fatty acids (% of total)	Control	PM5	PM10	PM15	PM20	Pooled SEM	*P* value		
							ANOVA	Linear	Quadratic
14:0	1.97	1.92	1.92	1.84	1.95	0.01	0.888	0.659	0.446
15:0	0.13	0.12	0.11	0.10	0.10	0.008	0.153	**0.003**	1.000
16:0	21.1	21.7	23.5	22.9	25.1	0.89	0.068	**0.002**	0.816
*17:0*	0.21	0.20	0.20	0.21	0.20	0.01	0.924	0.663	0.606
18:0	6.85^bc^	6.42^c^	7.00^abc^	8.03^ab^	8.22^a^	**0.27**	**0.003**	**0.001**	0.102
20:0	0.36	0.35	0.33	0.31	0.30	0.03	0.549	**0.043**	0.939
21:0	0.06	0.06	0.06	0.06	0.06	0.003	0.903	1.000	0.482
22:0	0.10	0.11	0.09	0.09	0.09	0.01	0.615	*0.099*	1.000
24:0	0.24^a^	0.22^ab^	0.23^ab^	0.21^ab^	0.20^b^	**0.01**	**0.029**	**0.002**	0.373
∑SFA	31.0^b^	31.1^b^	33.5^ab^	33.8^ab^	36.2^a^	**0.95**	**0.017**	**0.001**	0.401
16:1	3.50	3.74	3.69	3.35	3.50	0.18	0.573	0.472	0.441
17:1	0.12	0.11	0.09	0.10	0.09	001	0.250	**0.015**	0.452
18:1n-9	39.9	41.0	40.6	41.0	39.8	0.65	0.574	0.945	*0.083*
18:1n-7	3.03	3.06	2.95	2.81	2.89	0.12	0.565	0.102	0.852
20:1	1.70	1.69	1.47	1.51	1.33	0.14	0.325	**0.020**	0.866
22:1n-9	0.25	0.23	0.23	0.21	0.20	0.02	0.424	**0.027**	0.892
24:1	0.05	0.05	0.04	0.05	0.05	0.01	0.382	0.370	0.139
∑MUFA	48.6	49.8	49.1	49.1	47.9	0.54	0.208	0.154	**0.028**
18:2n-6	8.41	8.17	6.86	6.84	5.95	0.76	0.202	**0.006**	0.992
18:3n-3	2.07	1.98	1.92	1.9	1.82	0.12	0.638	*0.071*	0.940
18:4n-3	0.34	0.34	0.31	0.31	0.28	0.02	0.231	**0.008**	0.686
20:2n-6	0.66	0.63	0.56	0.58	0.53	0.06	0.564	*0.063*	0.739
20:3n-6	0.48	0.47	0.43	0.42	0.38	0.03	0.136	**0.003**	0.720
20:4n-6 (ARA)	0.22	0.21	0.21	0.19	0.19	0.01	0.312	**0.017**	0.771
22:2n-6	0.18	0.18	0.15	0.15	0.13	0.02	0.252	**0.011**	0.905
20:5n-3 (EPA)	1.04	1.09	1.02	0.93	0.86	0.08	0.308	**0.023**	0.295
22:4n-6	0.05	0.05	0.05	0.05	0.05	0.01	0.815	0.166	1.000
22:5n-3	0.36	0.36	0.32	0.33	0.29	0.05	0.778	0.176	0.729
22:6n-3 (DHA)	1.77	1.61	1.67	1.38	1.30	0.14	0.159	**0.006**	0.658
∑PUFA	15.6	15.1	13.5	13.1	11.8	1.22	0.245	**0.007**	0.895
PUFA/SFA	0.51	0.49	0.40	0.39	0.33	0.05	0.131	**0.003**	0.880
∑n-6	10.0	9.70	8.25	8.23	7.22	0.86	0.208	**0.007**	0.997
∑n-3	5.57	5.39	5.24	4.86	4.54	0.38	0.365	**0.017**	0.663
n6/n3	1.78	1.80	1.58	1.70	1.59	0.07	0.150	**0.038**	0.728
DHA/EPA	1.70	1.47	1.65	1.49	1.50	0.07	0.160	0.129	0.588

^*^Values with different superscripts in the same row are significantly different from each other (*P* < 0.05). Significant P values less than 0.05 are shown bold, whereas those significant trends between 0.05 and 0.10 are shown in Italic and underlined

Control (0% PM), PM5 (5% PM), PM10 (10% PM), PM15 (15% PM), and PM20 (20% PM), where PM denotes polychaete meal from *H. diversicolor*

*∑SFA*: total saturated fatty acids, *∑MUFA*: total monounsaturated fatty acids, *∑PUFA*: total polyunsaturated fatty acids, *∑n-6*: total n-6 polyunsaturated fatty acids, *∑n-3*: total n-3 polyunsaturated fatty acids, *(ARA)*: arachidonic acid, *EPA*: eicosapentaenoic acid, *DHA*: docosahexaenoic acid

**Table 10:** Fatty acid health indexes and estimated enzyme activities related to fatty acid metabolism in the liver*

	Control	PM5	PM10	PM15	PM20	Pooled SEM	P values		
							ANOVA	Linear	Quadratic
PI	40.5	39.1	37.2	34.2	31.5	2.92	0.257	**0.006**	0.610
Thioesterase	10.9	11.3	12.3	12.5	12.9	0.79	0.364	**0.017**	0.734
Elongase	0.33	0.30	0.30	0.35	0.33	0.01	*0.091*	0.219	0.294
∆9 desaturase (C16)	14.2^ab^	14.7^a^	13.6^abc^	12.7^bc^	12.3^c^	0.37	**0.005**	**0.001**	0.243
∆9 desaturase (C18)	86.2^ab^	87.3^a^	86.2^ab^	84.5^bc^	83.9^c^	0.46	**0.002**	**0.001**	**0.031**
∆9 desaturase (C16 + C18)	62.5^a^	62.9^a^	60.8^ab^	60.5^ab^	58.2^b^	0.77	**0.010**	**0.001**	0.156
∆5 + ∆6 desaturases (n6)	9.67	9.35	10.0	10.1	10.9	0.40	0.155	**0.008**	0.208
∆5 + ∆6 desaturases (n3)	60.3	60.7	60.9	57.5	57.4	1.11	0.115	**0.011**	0.248

^*^Values with different superscripts in the same row are significantly different from each other (*P* < 0.05). Significant P values less than 0.05 are shown bold, whereas those significant trends between 0.05 and 0.10 are shown in Italic and underlined

Control (0% PM), PM5 (5% PM), PM10 (10% PM), PM15 (15% PM), and PM20 (20% PM), where PM denotes polychaete meal from *H. diversicolor*

*PI*; peroxidation index

#### The fillet

The European seabass fillet was significantly affected by the dietary FM replacement with PM in terms of fatty acid profiles (P_*ANOVA*_ < 0.05), many of which were neither linearly nor quadratically correlated with dietary PM levels (Table 11). There were no significant effects on the total SFA, MUFA, and PUFA (*P* > 0.05) in response to the dietary inclusion of PM. However, individual Lc-PUFAs in fillets of European seabass, such as 20:4n-6 (ARA), and 20:5n-3 (EPA), showed a linear increase with PM levels (P_*LINEAR*_ < 0.05). This finding was partly confirmed by an increasing trend in ∆5 + ∆6 desaturases (n6) activities of the fillet of fish fed diets with gradually elevating PM concentrations in lieu of FM (Table 12).

**Table 11:** Fatty acid profiles of fillet of European seabass fed experimental diets for 49 days*

Fatty acids (% of total)	Control	PM5	PM10	PM15	PM20	Pooled SEM	P value		
							ANOVA	Linear	Quadratic
14:0	2.54	2.21	2.45	2.30	2.28	0.10	0.233	0.217	0.539
15:0	0.25	0.22	0.24	0.23	0.25	0.01	0.112	1.000	*0.052*
16:0	17.0^b^	21.2^a^	17.8^b^	19.3^ab^	19.1^ab^	0.59	**0.005**	0.419	0.265
*17:0*	0.24^ab^	0.23^b^	0.25^a^	0.25^ab^	0.26^a^	0.01	**0.013**	**0.004**	0.880
18:0	5.55^b^	10.7^a^	6.42^b^	7.82^ab^	7.86^ab^	0.90	**0.020**	0.658	0.320
20:0	0.21^a^	0.16^b^	0.21^a^	0.17^ab^	0.17^ab^	0.01	**0.010**	0.117	0.782
21:0	0.10^a^	0.07^b^	0.09^a^	0.09^ab^	0.09^ab^	0.004	**0.004**	0.469	*0.079*
22:0	0.18	0.15	0.17	0.16	0.16	0.01	0.102	0.210	0.286
24:0	0.27^a^	0.22^b^	0.25^ab^	0.23^ab^	0.22^b^	0.01	**0.021**	**0.008**	0.437
∑SFA	26.3^b^	35.2^a^	27.9^b^	30.5^ab^	30.4^ab^	1.39	**0.011**	0.583	0.292
16:1	3.62	3.18	3.63	3.44	3.43	0.14	0.217	0.782	0.688
17:1	0.08	0.10	0.08	0.09	0.10	0.01	0.679	0.403	0.858
18:1n9	30.0^a^	25.5^b^	28.5^ab^	27.5^ab^	27.5^b^	0.88	**0.050**	0.373	0.223
18:1n7	3.21	3.16	3.35	3.39	3.36	0.09	0.284	**0.037**	0.691
20:1	3.15^a^	2.49^b^	2.99^a^	2.80^ab^	2.92^ab^	0.10	**0.011**	0.744	0.105
22:1n9	0.33^a^	0.26^b^	0.30^ab^	0.29^b^	0.29^ab^	0.01	**0.011**	0.301	*0.081*
24:1	0.14	0.14	0.13	0.15	0.14	0.02	0.901	0.713	0.664
∑MUFA	40.5^a^	34.8^b^	39.0^ab^	37.7^b^	37.7^ab^	1.07	**0.039**	0.522	0.239
18:2n6	14.3^a^	12.1^b^	13.7^a^	12.9^ab^	13.2^ab^	0.33	**0.010**	0.327	0.166
18:3n3	2.48^a^	2.13^b^	2.39^ab^	2.36^b^	2.37^ab^	0.06	**0.020**	0.945	0.165
18:4n3	0.65	0.54	0.66	0.60	0.59	0.03	0.057	0.600	0.774
20:2n6	0.88^a^	0.77^b^	0.85^ab^	0.85^ab^	0.85^ab^	0.02	**0.036**	0.903	0.153
20:3n6	0.91^a^	0.69^b^	0.83^ab^	0.77^ab^	0.79^ab^	0.03	**0.007**	0.227	0.094
20:4n6 (ARA)	0.41^a^	0.49^a^	0.46^a^	0.48^a^	0.48^a^	0.02	**0.050**	**0.035**	0.101
22:2n6	0.44^a^	0.37^b^	0.43^a^	0.41^a^	0.41^a^	0.01	**0.001**	0.745	0.258
20:5n3 (EPA)	3.46	3.67	3.88	3.85	3.80	0.02	0.323	**0.038**	0.115
22:4n6	0.17	0.17	0.19	0.19	0.19	0.01	0.053	**0.003**	0.333
22:5n3	0.91	0.95	0.97	1.02	1.00	0.02	0.053	**0.001**	0.329
22:6n3 (DHA)	4.52	5.81	5.03	5.34	5.01	0.28	0.076	0.597	*0.068*
∑PUFA	29.1^ab^	27.7^b^	29.4^a^	28.8^ab^	28.7^ab^	0.33	**0.044**	0.917	0.867
PUFA/SFA	1.11^a^	0.79^b^	1.06^a^	0.95^ab^	0.95^ab^	00.05	**0.008**	0.505	0.346
∑n6	17.1^a^	14.6^b^	16.5^a^	15.6^ab^	15.9^ab^	0.36	**0.007**	0.387	0.161
∑n3	12.0	13.1	12.9	13.2	12.8	0.35	0.211	0.112	**0.036**
n6/n3	1.42^a^	1.12^b^	1.28^ab^	1.19^ab^	1.24^ab^	0.05	**0.023**	0.149	0.057
DHA/EPA	1.31^a^	1.59^a^	1.29^a^	1.39^a^	1.32^a^	0.06	**0.046**	0.504	0.303

^*^Values with different superscripts in the same row are significantly different from each other (*P* < 0.05). Significant P values less than 0.05 are shown bold, whereas those significant trends between 0.05 and 0.10 are shown in Italic and underlined

Control (0% PM), PM5 (5% PM), PM10 (10% PM), PM15 (15% PM), and PM20 (20% PM), where PM denotes polychaete meal from *H. diversicolor*

*∑SFA*: total saturated fatty acids, *∑MUFA*: total monounsaturated fatty acids, *∑PUFA*: total polyunsaturated fatty acids, *∑n-6*: total n-6 polyunsaturated fatty acids, *∑n-3*: total n-3 polyunsaturated fatty acids, *(ARA)*: arachidonic acid, *EPA*: eicosapentaenoic acid, *DHA*: docosahexaenoic acid

**Table 12:** Fatty acid health indexes and estimated enzyme activities related to fatty acid metabolism in the fillet of European seabass fed experimental diets for 49 days*

	Control	PM5	PM10	PM15	PM20	Pooled SEM	P values		
							ANOVA	Linear	Quadratic
PI	90.75	98.80	96.92	98.43	95.59	2.56	0.281	0.186	**0.037**
AI	0.49	0.48	0.48	0.48	0.48	0.008	0.816	0.383	0.549
TI	0.43^b^	0.52^a^	0.43^b^	0.46^ab^	0.47^ab^	0.015	**0.008**	0.798	0.550
h/H	2.99^a^	2.27^b^	2.85^a^	2.59^ab^	2.61^ab^	0.107	**0.007**	0.405	0.278
Thioesterase	6.71^b^	9.60^a^	7.31^ab^	8.42^ab^	8.46^ab^	0.548	**0.030**	0.295	0.382
Elongase	0.33^b^	0.51^a^	0.36^ab^	0.41^ab^	0.41^ab^	0.035	**0.039**	0.670	0.343
∆9 desaturase (C16)	17.58^a^	13.06^b^	17.01^ab^	15.18^ab^	15.28^ab^	0.860	**0.029**	0.483	0.409
∆9 desaturase (C18)	85.66^a^	72.73^b^	83.22^ab^	79.83^ab^	79.74^ab^	2.278	**0.023**	0.619	0.301
∆9 desaturase (C16 + C18)	62.01^a^	49.96^b^	59.51^a^	55.95^ab^	56.06^ab^	2.010	0.169	0.494	0.280
∆5 + ∆6 desaturases (n6)	8.31^b^	9.43^a^	8.71^ab^	9.39^a^	9.14^ab^	0.231	**0.028**	*0.069*	0.194
∆5 + ∆6 desaturase (n3)	78.19^b^	82.97^a^	80.36^ab^	81.17^ab^	80.54^ab^	0.921	**0.050**	0.375	*0.066*

^*^Values with different superscripts in the same row are significantly different from each other (*P* < 0.05). Significant P values less than 0.05 are shown bold, whereas those significant trends between 0.05 and 0.10 are shown in Italic and underlined

Control (0% PM), PM5 (5% PM), PM10 (10% PM), PM15 (15% PM), and PM20 (20% PM), where PM denotes polychaete meal from *H. diversicolor*

*PI* Peroxidation index, *AI* Atherogenicity index, *TI* Thrombogenicity index, *h/H* Hypocholesterolemic/hypercholesterolemic ratio

### Fish in: fish out ratio

**Fig. 1:**
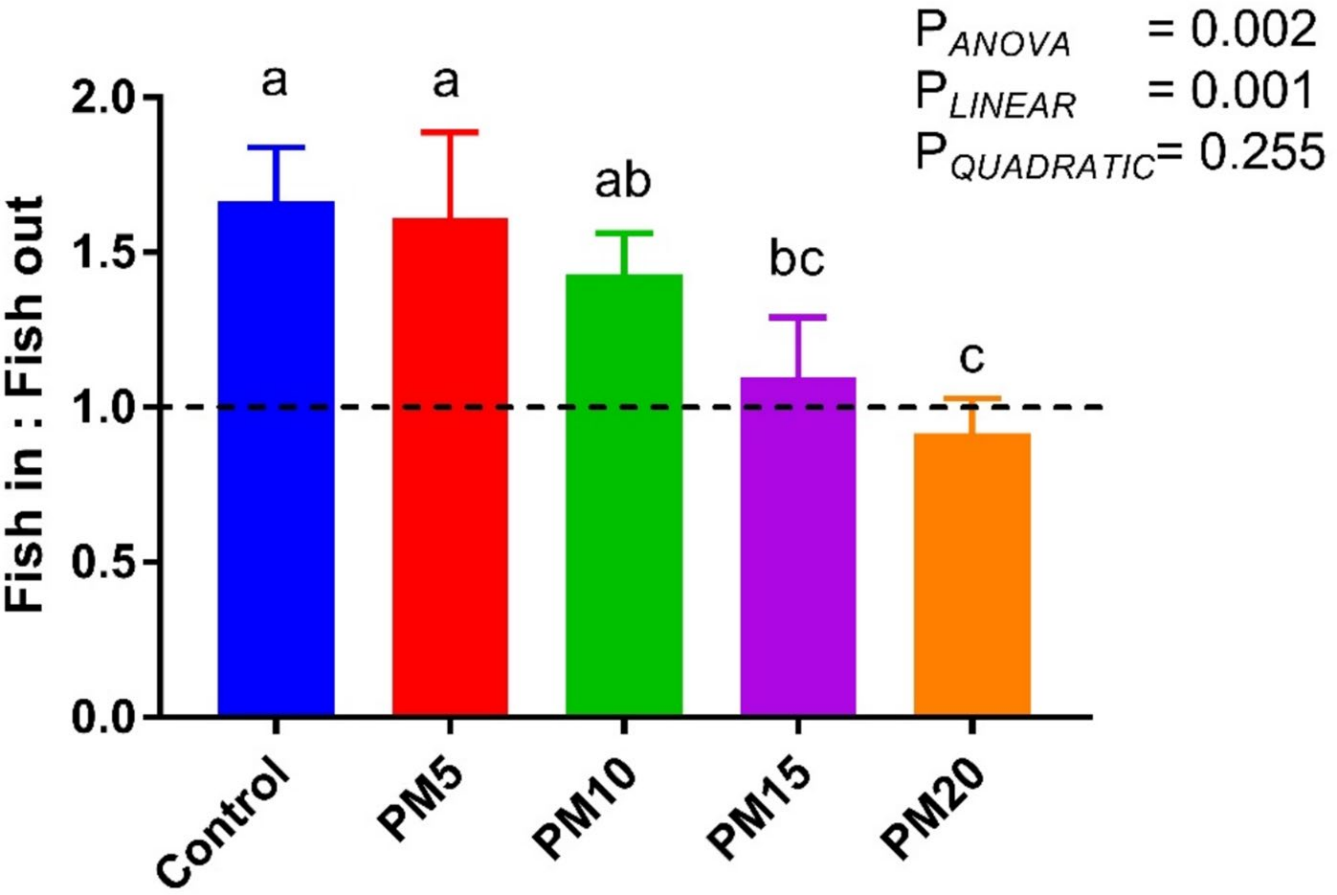
Fish in: fish out ratio in European sea bass fed increasing levels of dietary PM instead of FM. Bars with different letters are significantly different (*P* < 0.05)

The FIFO ratio of the dietary treatments is given in Fig. 1. Dietary incorporation of PM up to 20% significantly decreased FIFO compared with the control (P_*ANOVA*_ = 0.002), which displayed a significant linear decline with dietary PM levels (P_*LINEAR*_ = 0.001).

## Discussion

The PM used in the present study was lower in protein but comparable or higher in critical fatty acids such as ARA, EPA and DHA than the FM mixture. The nutrient composition of polychaetes changes depending on the species, origin (wild or culture) and their feed sources if being cultured or integrated to recycle the aquaculture wastes (Jayaseelan et al. 2021; Jerónimo et al. 2021a, b; Marques et al. 2018; Monteiro et al. 2024a; Pajand et al. 2017). *H. diversicolor* meal collected from nature in the present study contained about 60% protein, which is consistent with the levels of *H. diversicolor* from the wild (Mastoraki et al. 2023; Wang et al. 2019b), but higher than those maintained on aquaculture wastes, feeds, or biogas side streams (Wang et al. 2019a, b). The lipid level and the fatty acid profiles of *H. diversicolar* are rather variable depending on factors, such as environmental conditions, season and diets (García-Alonso et al. 2008; Jerónimo et al. 2021a, b; Lillebø et al. 2012; Marques et al. 2018; Wang et al. 2019a, b). The most abundant fatty acids of PM in the present study were 16:0, 18:0, 18:1n-9–7, 20:5n-3, and 22:6n-3, which are partly consistent with the literature findings (García-Alonso et al. 2008; Jerónimo et al. 2021a, b; Lillebø et al. 2012; Santos et al. 2016). The amino acid profiles of FM and PM adopted from the literature suggest that PM contains lower levels of some essential amino acids than FM, mainly tryptophan and threonine (37 and 63% of FM, respectively). However, when considered as a single protein source, the PM can cover all dietary requirements of essential amino acid concentrations for European seabass, except tryptophan, being consistent with the claim of Wang et al. (2019b), who evaluated the amino acid profile of polychaete reared on aquaculture wastes for the requirements of Atlantic salmon.

The use of polychaetes in fish diets has attracted some attention (Binh et al. 2008; Ende et al. 2017, 2018a, b; Gao et al. 2024; Kals et al. 2017; Monteiro et al. 2024b). However, the majority of these studies evaluated polychaetes as whole prey rather than as a processed feed ingredient. When polychaete was used singly for feeding common sole, the growth performance significantly improved compared with fish fed only commercial dry pellet or pellets with polychaete extract (Ende et al. 2017; Kals et al. 2017). Growth and reproductive performance also improved in male black tiger shrimp (*Penaeus monodon*) fed polychaete, *Perinereis nuntia* (Leelatanawit et al. 2014). Studies dealing with polychaete as a feed ingredient show that dietary PM can sustain growth and nutrient utilization performance in several species. For example, replacement of fish meal with PM (from *Nereis virens*) in the diets of shrimp (*Litopenaeus vannamei*) resulted in comparable growth and feed utilization performance, and survival rate (Lupatsch 2014). In European seabass, inclusion of 10% PM from *A. virens* supported similar growth to FM-fed fish (Stabili et al. 2019; Monteiro et al. 2024b). Freeze- or oven-dried PM from *N. japonica* yielded similar growth and feed efficiency responses in juvenile tiger puffer (*Takifugu rubripes*) when included up to 9% (Gao et al. 2024). Consistent with these studies, growth rate and feed conversion efficiency of European seabass in the present trial were not negatively affected by PM inclusion.

Feed intake tended to increase with the increase of dietary PM levels in the present study (P_*LINEAR*_ = 0.068), which is consistent with previous studies showing that polychaetes have feeding stimulants, particularly free amino acids (Ende et al. 2009; Kals 2017; Pinandoyoa et al. 2021; Gao et al. 2024). This may have likely played a role in increasing feed consumption with dietary PM levels in the present study. However, this trend was not observed in nutrient intakes, gains, and retentions, except lipid gain and retention, which showed a significant linear increase with the levels of PM. Monteiro et al. (2024b) also reported no negative effects on nitrogen or energy balances in seabass fed PM diets. We also did not detect any significant changes in the nutrient losses variables among dietary treatments, suggesting that increased level of dietary PM did not cause adverse environmental impacts.

ADCs of dry matter, protein, energy, and organic matter linearly increased in the present investigation, indicating improved nutrient absorption when PM partially replaced FM in European seabass diets. The present findings are not consistent with those of Monteiro et al. (2024b), who recorded similar ADC values in European seabass fed up to 10% PM (*A. virens*) and Mastoraki et al. (2023), who found lowered ADCs for dry matter and energy in meager (*Argyrosomus regius*) above 10% PM incorporation. Studies on shrimp (*L. vannamei*) and tiger puffer have also reported variabilities in ADCs and enzyme activities among dietary polychaete sources (Lupatsch 2014; Gao et al. 2024). The discrepancies between the present findings and those of the literature may reflect differences in polychaete species, processing conditions, diet formulations, and species-specific digestive physiology. In this study, the linear improvement may be attributed to the highly digestible marine protein, lipid profile of *H. diversicolor* (see below for improved ADCs for saturated fatty acids), and the absence of anti-nutritional factors, suggesting more efficient digestive enzyme access to the nutrients. Yet, more studies are needed to get deeper insight into the nutrient availabilities of polychaetes in fish diets.

ADCs of ∑SFA increased quadratically with the levels of dietary PM, mainly due to 16:0, 18:0, 20:0, and 24:0, reflecting the high proportion of readily digestible marine lipids in *H. diversicolor*. Monounsaturated fatty acid ADCs did not change significantly with the treatments, although a quadratic effect was observed in 18:1n-7 and 22:1n-9. The ADCs of individual n-6 and n-3 PUFAs were comparable among the diets, but ADC of Lc-PUFAs linearly increased with dietary PM. There is no available data related to the fatty acid digestibility of polychaetes in finfish. However, our unpublished data from a companion study in gilthead seabream align with those in the results of our present study, suggesting that gradual inclusion of dietary PM in European seabass in place of FM positively affected the ADCs of saturated fatty acids and Lc-PUFAs.

The whole body moisture levels linearly decreased with the concentrations of PM, whereas an opposite linear increase was observed in the protein and lipid. A similar inverse relationship between body moisture and protein or lipid deposition has been widely reported in fish, reflecting greater nutrient deposition as body energy reserves increase (Shearer 1994). Accordingly, the observed changes in the present study likely reflect improved ADCs of dry matter, protein, organic matter, and energy. The lipid deposition appears to have occurred primarily in muscle tissue rather than in visceral fat stores, considering that VSI values were unaffected among treatments. The proximate composition of gilthead seabream was unaffected by the same source of PM, being inconsistent with the present results (our unpublished data). Likewise, adding PM from *A. virens* and *N. japonica* to diets of European seabass and tiger puffer to replace FM did not change any of the whole body nutrient composition (Gao et al. 2024; Monteiro et al. 2024b). Dietary freeze-dried PM in the diets of Pacific white shrimp resulted in comparable whole-body nutrient content (Lupatsch 2014). Obviously, the influence of dietary PM on the whole body proximate composition varies among aquaculture species and appears to change depending on the species of polychaete used.

The liver fatty acid profile of fish was highly sensitive to changes in dietary PM levels in the present study. The ∑MUFAs, for instance, linearly increased due to the increase in 16:0 and 18:0. A comparison of liver fatty acid profiles between wild and cultured female Senegalese sole (*Solea senegalensis*) revealed that the wild individuals had higher ∑MUFAs than those cultured, which was attributed to polychaetes, one of the main food items in the wild (Norambuena et al. 2012). Despite PM's higher PUFA content, hepatic PI declined linearly with increasing PM inclusion (P_*LINEAR*_ = 0.006), suggesting reduced tissue FA unsaturation and peroxidation susceptibility. The PI reflects the degree of FA unsaturation, with higher values linked to elevated Lc-PUFA content, which increases oxidative stress via reactive oxygen species (Almaida-Pagán et al. 2015; Gourtay et al. 2020). Thioesterase activity increased linearly in the liver (P_*LINEAR*_ = 0.017). Thioesterases (e.g., acyl-CoA thioesterases, ACOTs) hydrolyze acyl-CoA to free FAs, regulating FA partitioning for beta-oxidation, synthesis, or export (Tillander et al. 2017). The linear increase in thioesterase activity may also indicate an enhanced lipogenic capacity or an adaptation to lipotoxicity in fish fed increasing PM (Zhang et al. 2012). This observation may serve as a compensatory mechanism for dietary shifts, as also implied by higher ∆9 desaturase activity at lower PM levels. Major MUFAs of the liver, such as 16:1 and 18:1n-9, seemed unresponsive to dietary changes with increased dietary PM levels, being consistent with the findings in Senegalese sole (Norambuena et al. 2012). However, ∑n6 and its members (except for 20:2n-6 and 22:4n-6) and ∑n-3 and its members (excluding 18:3n-3 and 22:5n-3) in the liver linearly reduced along with the dietary PM levels. This reduction was reflected in a decreasing trend in ∑PUFA and PUFA/SFA. A linear decrease in DHA/EPA ratios further highlights a shift in the n-3 PUFA balance, which might impact the membrane structural functions of these fatty acids. The decline in ∆5 + ∆6 desaturase activity for n-3 specifically at higher PM levels underscores a reduced conversion efficiency of precursor PUFAs into bioactive long-chain derivatives like DHA. The findings suggest that dietary PM inclusion may compromise PUFA bioavailability, enhancing hepatic lipogenesis, as reflected in higher SFA synthesis and reduced susceptibility to oxidation with lower PI values. However, whether longer-term feeding of high PM levels leads to hepatic steatosis or alters antioxidant requirements warrants further investigation.

The inclusion of PM exerted a significant influence on the fatty acid composition and lipid quality indices of European seabass fillets, but not in a clear dose-dependent manner, as observed in European seabass fed diets including up to 10% PM from *A. virens* (Monteiro et al. 2024b). For instance, ∑SFA, ∑MUFA, and their associated enzyme activities displayed similar levels across the dietary levels of PM. However, PI values displayed a quadratic trend with the concentrations of PM, which indicates muscle tissue incorporates more PUFAs (e.g., EPA) for structural integrity, enhancing nutritional quality without excessive peroxidation (Hasan et al. 2024; Ulbricht and Southgate 1991). The AI, TI, h/H and ∑n-6 fatty acids exhibited comparable values among the treatments, with some exceptions, such as 20:4n-6 and 22:4n-6 that increased steadily with the dietary levels of PM. The increasing trends in these fatty acids cannot be explained by a reflection of dietary fatty acid profiles in the fillet, considering their levels in the diets were comparable, but may be attributed to an increased tendency in ∆5 + ∆6 desaturases (n-6) activity with the PM levels (P_*LINEAR*_ = 0.069). Dietary replacement of FM with PM also increased the levels of 20:5n-3 and 22:5n-3, but not 22:6n-3, an increased activity of ∆5 + ∆6 desaturase (n-3), encoded by fads2, which introduces double bonds to synthesize Lc-PUFAs (e.g., 18:3n-3 to 20:5n-3) (Tocher 2015). The results of fillet n-3 fatty acid profiles in the present study are partly consistent with those of Glencross et al. (2024b), who claimed that the utilization efficiencies of all fatty acids can be considered constant across different intake levels, except DHA, which is less well retained as intake increases. There were also tissue-dependent differences between liver and muscle in fatty acid profiles and enzyme activities in the current study. The liver, as metabolically the most active organ, responded to dietary PM inclusion by adjusting fatty acid synthesis, elongation, desaturation, and oxidation pathways, as reflected in changes in ∑PUFA, PUFA/SFA ratios, and indices of thioesterase and desaturase activities (Anedda et al. 2023; Ferosekhan et al. 2020). In contrast to hepatic responses, the muscle tissue showed a more limited response, with fillet fatty acid composition remaining relatively stable across dietary PM levels. This finding is consistent with previous studies demonstrating greater metabolic plasticity of liver lipids compared with muscle, where fatty acids, especially those associated with structural lipids, exhibit lower turnover and reduced dietary responsiveness (Torstensen et al. 2004; Böhm et al. 2014). Although we assessed lipid metabolism indirectly via fatty acid composition and calculated desaturase/elongase indices in the tissues, future studies should incorporate direct metabolic and health biomarkers to better characterize hepatic and whole-body responses.

The FIFO ratio is an important criterion in terms of the use of marine ingredients and resulting feed utilization performance (Tacon and Metian 2008). In the present study, substituting FM with PM led to a marked reduction in FIFO from 1.70 in the control diet to 0.92 in the PM20 diet. This value falls below the commonly cited sustainability threshold for marine resource use (Majluf et al. 2024). This further boosts the potential of PM as a sustainable dietary alternative to marine ingredients in the diets of European seabass.

## Conclusions

The results of the present study clearly show that PM is a valuable ingredient that replaces 50% of FM and partly FO in the diets of European seabass. The inclusion of PM in diets of up to 20% supports comparable growth and feed utilization without jeopardizing fillet fatty acid profiles for human food. The improved nutrient digestibility and lipid retention suggest that PM provides a highly digestible marine-derived-animal protein and lipid source, including Lc-PUFAs. The inclusion of PM induced some moderate alterations in hepatic lipid metabolism, slightly increasing SFA content and reducing hepatic PUFA/SFA ratio, but the long-term physiological implications of hepatic responses require further study. Importantly, a significant environmental benefit was evidenced by about 50% reduction in the FIFO ratio at 20% PM inclusion, underlining the sustainability potential of PM as a feed ingredient. Overall, these findings support the potential of PM as a nutritionally viable and environmentally sustainable alternative marine ingredient. However, future studies should extend the present findings by incorporating longer-term rearing under larger settings and direct metabolic and health biomarkers to get more insights into physiological and sustainability implications of PM inclusion.

## Data Availability

Data availability is possible when requested.
